# A Critical Review on Metallic Glasses as Structural Materials for Cardiovascular Stent Applications

**DOI:** 10.3390/jfb9010019

**Published:** 2018-02-27

**Authors:** Mehdi Jafary-Zadeh, Gideon Praveen Kumar, Paulo Sergio Branicio, Mohsen Seifi, John J. Lewandowski, Fangsen Cui

**Affiliations:** 1 Institute of High Performance Computing, A*STAR, Singapore 138632, Singapore; vijayagpk@ihpc.a-star.edu.sg; 2 Mork Family Department of Chemical Engineering and Materials Science, University of Southern California, Los Angeles, CA 90089-0241, USA; branicio@usc.edu; 3 Department of Materials Science and Engineering, Case Western Reserve University, Cleveland, OH 44106, USA; mohsen.seifi@case.edu (M.S.); jjl3@case.edu (J.J.L.)

**Keywords:** metallic glass, fatigue, additive manufacturing, stent, nitinol, heart valve, finite element analysis

## Abstract

Functional and mechanical properties of novel biomaterials must be carefully evaluated to guarantee long-term biocompatibility and structural integrity of implantable medical devices. Owing to the combination of metallic bonding and amorphous structure, metallic glasses (MGs) exhibit extraordinary properties superior to conventional crystalline metallic alloys, placing them at the frontier of biomaterials research. MGs have potential to improve corrosion resistance, biocompatibility, strength, and longevity of biomedical implants, and hence are promising materials for cardiovascular stent applications. Nevertheless, while functional properties and biocompatibility of MGs have been widely investigated and validated, a solid understanding of their mechanical performance during different stages in stent applications is still scarce. In this review, we provide a brief, yet comprehensive account on the general aspects of MGs regarding their formation, processing, structure, mechanical, and chemical properties. More specifically, we focus on the additive manufacturing (AM) of MGs, their outstanding high strength and resilience, and their fatigue properties. The interconnection between processing, structure and mechanical behaviour of MGs is highlighted. We further review the main categories of cardiovascular stents, the required mechanical properties of each category, and the conventional materials have been using to address these requirements. Then, we bridge between the mechanical requirements of stents, structural properties of MGs, and the corresponding stent design caveats. In particular, we discuss our recent findings on the feasibility of using MGs in self-expandable stents where our results show that a metallic glass based aortic stent can be crimped without mechanical failure. We further justify the safe deployment of this stent in human descending aorta. It is our intent with this review to inspire biodevice developers toward the realization of MG-based stents.

## 1. Introduction

Crystalline solid-state materials with all types of chemical bonds, i.e., van der Waals, hydrogen, covalent, ionic, and metallic, can be made amorphous by various techniques [1,2]. For historical reasons, however, only an amorphous material obtained by rapid quenching from the liquid state is called glass [2]. In this regard, “amorphous” refers to the lack of translational symmetry in their atomistic configuration (see Section 4 for more details). Indeed, the process of rapid amorphisation of a liquid, i.e., vitrification, is the major approach to obtain a glass. The corresponding cooling rate strongly depends on the employed technique and material. Hence, in the current review, we use the term “metallic glass” (MG) to refer to an amorphous solid alloy obtained by the rapid quenching of its melts. The combination of metallic bonds and amorphous structure in MGs provides fascinating and extraordinary structural and functional properties superior to their crystalline counterparts. Thus, a great amount of attention and effort has been devoted to understand MGs and exploit their superior properties for technological development, including health and biomedical applications. 

There are numerous review articles [1,3,4,5,6,7] and textbooks [8,9] on the general aspects of MGs or their specific features, such as functional/physical properties [10], mechanical properties [11,12,13], and corresponding small/nano-sized effects [14]. Furthermore, specialized reviews have discussed their shear banding phenomena [15,16], and the relationships between their atomistic structure and properties [17,18]. More recently, certain attention has been paid to the biocompatibility and medical application of MGs (see Section 6 and [19,20,21]) while, in the biomedical community, the major focus is on the chemical properties (corrosion resistance) and biocompatibility of MGs and, to the best of our knowledge, their mechanical behaviour for biodevice applications has not received proper attention. Particularly, while it is widely acknowledged that MGs are very high strength materials, their severe localized plastic deformation upon yield at ambient conditions has been overlooked. For example, recently, there has been increasing attention towards employing metallic glass as a promising material for cardiovascular stent applications [21]. However, as we will discuss in detail in this article, due to such a severe localized plastic deformation, application of MGs in balloon-expandable stents is constrained, and certain caveats must be taken into account to design other devices, such as self-expandable stents. To clarify these important concepts, here, we first try to provide a brief yet comprehensive review of metallic glasses from the materials science point of view. Our major focus is on the formation of conventional and bulk metallic glasses, their advanced manufacturing techniques, structure and mechanical properties, and the interconnection of these issues. Then, we explain the current categories of cardiovascular stents, their mechanical requirements, and the popular materials used in each category. These discussions shed light on how exploiting the functional and structural properties of MGs can enhance the performance of the next generation of stents and other bio-devices. We finalize this article by reviewing our recent findings on the feasibility of developing MG-based cardiovascular stents.

## 2. Conventional Metallic Glasses vs. Bulk Metallic Glasses

Generally, glasses are formed through a vitrification process, which is quenched quickly from their liquid state [22]. On the one hand, if the quench rate is slow enough, the system kinetics allows the crystalline phase to nucleate below the melting temperature, *T_m_*, and the liquid is transformed to a crystalline solid. On the other hand, if the quench rate of the liquid is sufficiently high, the nucleation of the crystalline phase will be impeded. Consequently, the liquid can be cooled below *T_m_* without crystallization. In this case, the liquid is in the supercooled state, which is a metastable state as compared with the crystalline state. Upon further cooling of the supercooled liquid, its viscosity increases. Eventually, at a certain temperature below *T_m_*, the viscosity of the supercooled liquid reaches a value around 10^12^ Pa.s, i.e., 10^13^ Poise, which is almost 14–15 orders of magnitude higher than the viscosity of liquid at *T_m_* [23]. By definition, this temperature is called the glass transition temperature, *T_g_*, at which the atomic structure of the supercooled liquid remains almost stationary [23]. In other words, at *T_g_*, the mobile melt turns to a rigid glass [22]. In the rapid quenching processes, the “critical cooling rate”, *R_c_* is the slowest cooling rate that allows the material to avoid crystallization. Indeed, *R_c_*, is a measure of the glass-forming ability (GFA). That is, an alloy with a lower *R_c_* has a better GFA than an alloy having higher *R_c_*. On the other hand, the cooling rate and sample dimensions (e.g., its thickness) are closely related. Hence, *R_c_* also corresponds to the critical thickness, *d_c_*, which is the maximum thickness that a glassy sample could be obtained from a given alloy. 

Compared to the traditional mineral (silicate) or organic glass forming materials, pure metals and ordinary metallic alloys readily crystallize upon quenching. Therefore, formation of glassy metals is rather difficult compared to traditional non-metallic glasses. This is why formation of the first metallic glass from Au–Si alloy required a very fast cooling rate of 10^6^ K/s, and very small dimensions of 50 μm [24]. For many alloys and elemental (pure) metals, a fast cooling rate on the order of 105 to 1010 K/s is required to generate a glass. Such metallic glasses are called conventional MGs [22]. A variety of MG-forming alloys have been categorized in several manners [25]. For example, MGs can be labelled based on their dominant alloying component such as Zr-based, Fe-based, Mg-based, Al-based, etc. Additionally, they can be categorized as metal–metal or metal–metalloid types to emphasize the nature of their constituent elements. Moreover, various glass forming alloys can also be classified based on their GFA, i.e., slower critical cooling rate, *R_c_*, or equally, higher critical thickness, *d_c_*.

In contrast with conventional MGs which have critical thicknesses in the sub-millimetre range, alloys with good GFA, which allows them to be casted in samples with at least 1 mm thickness, are called bulk metallic glasses (BMGs) [26]. The first BMG was produced from the Pd–Cu–Si alloy with a cooling rate of 10^3^ K/s [26], while the term ‘‘bulk’’ was arbitrarily defined as the millimetre scale. Later on, Turnbull et al. [27,28] obtained a Pd–Ni–P BMG with a centimetre thickness and cooling rate of only 10 K/s. They had highly purified the melt to minimize the possibility of heterogeneous nucleation. However, the expensive Pd-based ternary alloys mainly remained as laboratory materials.

Inoue et al., in the late 1980s, discovered new multicomponent alloy systems of common metallic elements having low critical cooling rates [29,30]. By systematic investigation of the GFA in ternary systems of Fe and Al metals with rare-earth elements, they obtained exceptional BMGs such as La–Cu–Al samples with thicknesses of several millimetres [30]. Based on these findings, quaternary and quinary BMGs, such as La–Al–Cu–Ni and La–Al–Cu–Ni–Coat, were synthesized with low cooling rates of less than 100 K/s. Further research revealed similar BMG forming alloys designed by partially replacing the rare-earth metals with Mg (an alkaline metal), such as Mg–Cu-Y, Mg–Ni-Y, etc., [31], along with a family of multicomponent Zr-based BMGs, such as Zr–Cu–Ni, Zr–Cu–Ni–Al) [32]. It is noteworthy that Inoue [33] and Takeuchi et al. [34] have reviewed and provided a sophisticated classification of BMGs, and suggested several empirical rules to design new BMG forming alloys [34,35]. Briefly, Inoue’s empirical rules postulate that BMG forming systems, i.e., those with high GFA, are multicomponent systems in which the difference in atomic radii exceeds 12% and have a good solubility with high negative heat of mixtures. In addition to the continuous search for novel BMG forming alloys, the development of innovative manufacturing techniques is also an active and persisting research topic, which is further discussed in the next section. 

## 3. An Innovative Processing Route of Metallic Glasses: Additive Manufacturing 

**Figure 1:**
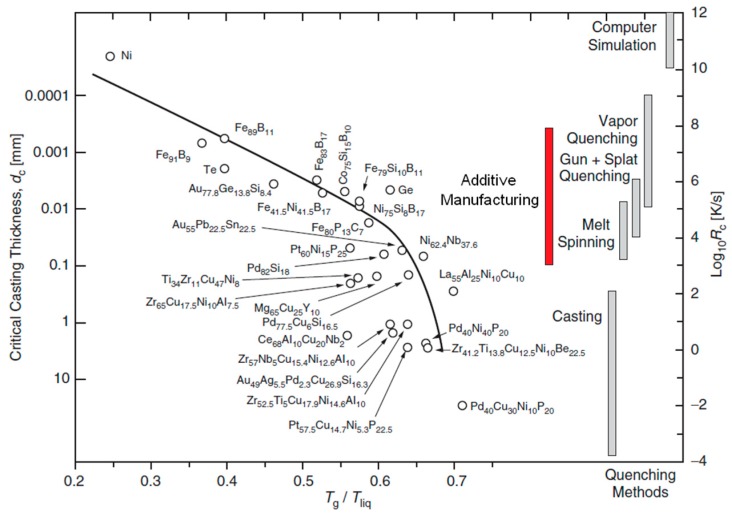
The critical cooling rate, *R_c_*, and the critical casting thickness, *d_c_*, for various glass-forming metallic alloys vs. their reduced glass transition temperatures, *T_g_*/*T_liq_*, and the correlation with a variety of quenching techniques [2]. The highest critical cooling rate needed for single element metals to amorphize can only be generated using special nanoscale experimental techniques or by using computer simulations. By adjusting the process parameters in additive manufacturing (AM) techniques, it would be possible to achieve an effective cooling rate on the order of 10^3^ to 10^8^ K/s. The layer-wise nature of fabrication in the AM technique and the corresponding highly-effective cooling rate would provide n opportunity to manufacture MG-based components with complex shapes and desirable thicknesses using simpler alloy compositions (reproduced with permission [2]).

To manufacture a variety of MGs/BMGs, melting and quenching techniques have been widely explored and developed in the last few decades. Figure 1 shows a variety of metallic systems according to their critical cooling rate, *R_c_*, and critical thickness, *d_c_*, against their reduced glass transition temperature, *T_g_/T_liq_*, where *T_liq_* is their melting temperature [36]. This diagram also shows a variety of processing techniques and the range of their effective cooling rate. The traditional processing routes, such as casting, melt spinning, or gas atomization have intrinsic limitations to manufacture metallic glass (MG) structures with geometrical complexities and varying dimensions, such as stents. On the other hand, recent advances in additive manufacturing (AM) techniques have been tantalizing for fabrication of complex MG-based structures owing to their rapid effective quenching rate [37,38,39,40,41,42,43,44,45,46,47,48,49,50,51,52,53,54,55,56]. 

Commonly, AM methods are applied to produce complex geometries and components from a variety of conventional metallic alloys and polymers as described by Apple US patent [57]. On the other hand, powder bed fusion (PBF) techniques like selective laser melting (SLM) [38,43,45,51,52,54,55] or selective electron beam melting (SEBM) [58,59], in addition to directed energy deposition (DED) methods, such as laser engineered net shaping (LENS) [48,50] have high potential to overcome the limitations of traditional methods for MG processing. Early works investigated the application of electron beam (EB) welding to join Ti alloy plate or powder to Zr-based MG plate [44,56]. Basically, the layer-wise construction of a part/component that occurs by localized melting accomplished by fast scanning of a high-power source, such as a laser or electron beam, breaks down the melting/quenching process into several steps, each of which is sufficiently fast to guarantee glass-formation. To this end, proper selection of alloy powder and optimization of processing parameters are crucial [41,49,52,57]. 

Multiple factors, such as processing parameters (source power, scan speed, hatch spacing, laser energy density, laser spot size, etc.) [45,49,51,53,54], powder/substrate microstructure (crystalline or amorphous), and chamber/substrate temperature influence the quality of the resulting amorphous phase. For example, a higher substrate temperature could lead to a stronger interface bonding between the MG and substrate due to its direct impact on the cooling rate and thermal history [43]. Importantly, the effective cooling rate depends on the scanning speed of the laser beam, as well as several other processing factors such as the powder composition and temperature gradient between the melt pool and the substrate. In particular, by adjusting the process parameters, it would be possible to achieve an effective cooling rate in the range of 10^3^ to 10^8^ K/s during SLM process [42] (see Figure 1 to compare the effective cooling rate of AM techniques with those of traditional processes). It is noteworthy that an effective cooling rate of ~10^3^ K/s [55] is plausible for a Zr-based BMG, which has a critical cooling rate of ~10^2^ K/s or lower [8] (also see Figure 1 for critical cooling rate of Zr-based MGs). A proper processing regime and conditions can produce a desirable high density [45]. Moreover, it has been demonstrated that the amorphous phase is achievable on both amorphous and crystalline substrates owing to the inherent high cooling rate of the laser deposition process [48,50]. Powder size can also significantly affect the quality and uniformity of the final amorphous phase [47]. Larger powder size could induce low thermal stability leading to undesirable severe crystallization, while smaller powder size could offer a monolithic amorphous structure [47]. 

In summary, the additive manufacturing (AM) of MGs has recently received considerable attention as a promising technique to manufacture complex geometries with a desirable thickness, which was not possible otherwise. AM technologies could be very beneficial to fabricate thin and complex biological devices, such as cardiovascular stents [60,61] and devices [62,63], especially using metallic glasses. More specifically, in relation to vascular stents, when ill-fitted stents move within the artery, the very purpose of the vascular surgery could be defeated. In such cases, there may be a need for physicians to open up the blocked stent again, or bypass it with a vascular graft which, in turn, is a costly and risky process. Poor fit can be a result of geometric constraints in the patient’s vessel, such as an increased curvature resulting in secondary flow profiles which can disturb the blood flow [64], causing traditionally-fabricated stents to fail. This is of particular importance in patients who have premorbid conditions where the use of blood thinners is avoided, which are commonly administered to patients in whom stents have been deployed [65,66,67,68]. Thus, by additive manufacturing, customized stents that have the same geometric (structural) and biologic (functional) requirements of the patient’s blood vessel the above complications can be mitigated.

## 4. Structure of Metallic Glasses

**Figure 2:**
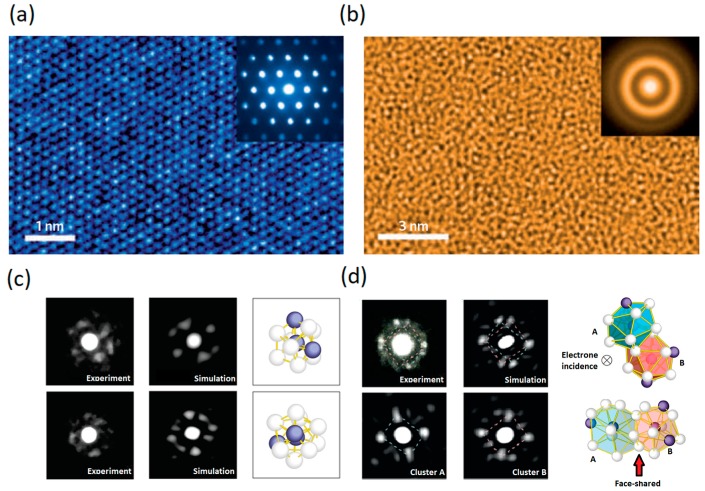
(**a**,**b**) Atomic-level structures of crystalline metals vs. metallic glasses [69]. (**a**) High-resolution TEM image of crystalline low-carbon steel showing well-defined and long-range order packing of lattice planes. (Inset) The corresponding selected-area electron diffraction (SAED) pattern showing sharp crystalline spots; and (**b**) high-resolution TEM images of a Zr_67_Ni_33_ metallic glass, illustrating a prototypical maze-like pattern in MGs. (Inset) The corresponding SEAD pattern of MG exhibits diffusive haloes; and (**c**,**d**) using nanometer diameter electron beam diffraction, short- and medium-range order (SRO and MRO) in MGs become evident [70].

The exact atomic configuration of metallic glasses has been an intriguing mystery for decades since their discovery. The transmission electron microscope (TEM) images shown in Figure 2a,b demonstrate the difference between the atomistic packing of a prototypical crystalline alloy, and that of a prototypical metallic glass, respectively. In the crystalline alloy (Figure 2a), the TEM image clearly resolves the long-range order (LRO) packing, i.e., translational symmetry, of atoms within well-defined crystal planes. However, even such high atomic resolution images cannot reveal any discernable structure or distinguishable order in the metallic glass (Figure 2b); instead, it just reveals a “maze-like pattern” [69]. The result of electron scattering patterns, the so called selected-area electron diffraction (SAED) pattern, is shown for both crystalline and glassy alloys in the inset of Figure 2a,b, respectively. The SAED pattern for the crystalline material shows a set of sharp and bright spots, each of which is the result of strong interference peaks that arise from long-range ordering (LRO) of similar crystalline planes. In contrast, and in the absence of LRO in the glassy material, the interference peaks are smeared out, leaving only a diffraction pattern, as diffuse halos, as shown in the inset of Figure 2b. While such a pattern of diffusive halos is a signature of metallic glasses, it does not provide further information on details of their atomic structure.

Recently, the structure of MGs has been widely modelled [18,71,72,73] as comprising interpenetrating quasi-equivalent clusters, i.e., coordination polyhedral, as initially had been suggested by Kasper and Frank, to model the structure of complicated alloys [74,75]. Based on this model, each atom in the alloy is surrounded by a preferred number of neighbouring atoms which are arranged in its nearest neighbour shell with a preferred chemical composition. These local atomic arrangements at the length scale of nearest neighbour shell define the short-range order (SRO) motifs of the MGs [76,77]. These SRO clusters then connect, and even overlap, with each other throughout the whole glassy alloy and form a variety of configurations beyond the nearest-neighbour shell, on a scale of a few to almost 10 Å, which is known as medium-range order (MRO).

The SRO- and MRO-based model of the MG structure has been strongly supported by realistic computer modelling based on first principles calculations and large-scale molecular dynamics simulations [73,77,78,79,80,81,82,83,84], and was successfully applied to explain experimental observations [82,84,85,86]. It is also noteworthy that a “direct observation” of SRO and MRO structures in MGs have been recently reported in nanobeam electron diffraction (NBED) experiments by exploiting a subnanometer diameter electron beam to acquire the diffraction patterns from a nanoscale regions of the MGs [70], as shown in Figure 2c,d. 

Recent advances in understanding the atomic structure of metallic glasses using advanced experimental and computational techniques provides the opportunity to shed light on the structure–property relationships in this class of materials. The role of the local structure in glass formation and dynamical arrest of the molten alloys during the vitrification process has been comprehensively reviewed in [72]. Cheng and Ma [18] provide a comprehensive review on the tremendous amount of work intended to reveal the atomistic structure of MGs and the structural origin of their properties. In the next section, we explain how the structural difference between MGs and their crystalline counterparts provide them their extraordinary mechanical properties such as high strength, high resilience, yet localized plastic deformation upon yield. The elastic and plastic properties of MGs is the focus of this article, as it is essential to exploit them in implantable medical devices, especially cardiovascular stents.

## 5. Mechanical Properties

The primary nature of atomic bonds in both glassy and crystalline metallic alloys is similar, which are mainly “metallic bonds”. However, regardless of the similarity in the nature of atomic bonds, metallic glasses exhibit mechanical properties significantly different in comparison with conventional crystalline alloys, as explained below. 

### 5.1. Strength Close to the Theoretical Limit

Metallic glasses have remarkably higher yield strength and hardness than their crystalline counterparts, which are much closer to the theoretical limit of the materials. Additionally, MGs have exceptionally high elastic limit and resilience, allowing them to store a substantially higher amount of elastic energy per unit volume compared to their crystalline polymorphs [5]. The underlying physics of such superior mechanical properties in MGs is attributed to their amorphous structure, that is, the lack of translational symmetry in their atomic structure. Due to their disordered atomic structure, MGs clearly do not have well-defined crystallographic slip systems and dislocation-mediated yield mechanisms. Hence, their deformation mechanisms are fundamentally different as compared to crystalline metals. In the absence of dislocation mediated mechanisms, MGs exhibit high yield strengths close to the theoretical limit as compared to their crystalline counterparts, and around 2.6% of elastic strain at room temperature [87,88]. However, they suffer from a lack of tensile ductility at room temperature which is an important drawback in their widespread structural application. These concepts are further explained in the next sections.

### 5.2. Elastic Properties: High Resilience

**Figure 3:**
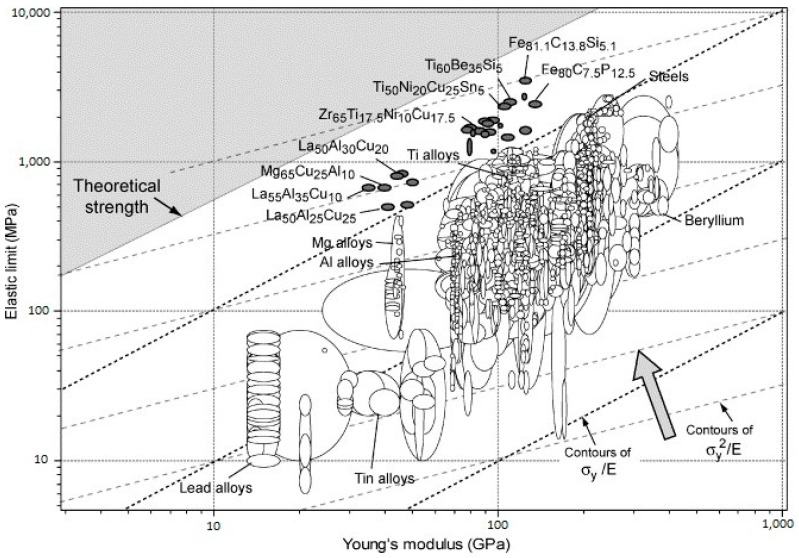
Elastic limit (yield stress) *σ_y_* versus the Young’s modulus, *E*, for about 1500 conventional alloys, metal-matrix composites, and BMGs (annotated with compositions in at %). The contours are for the elastic strain limit (*σ_y_/E*) and resilience (*σ_y_*^2^/*E*) (reproduced with permission [91]).

**Figure 4:**
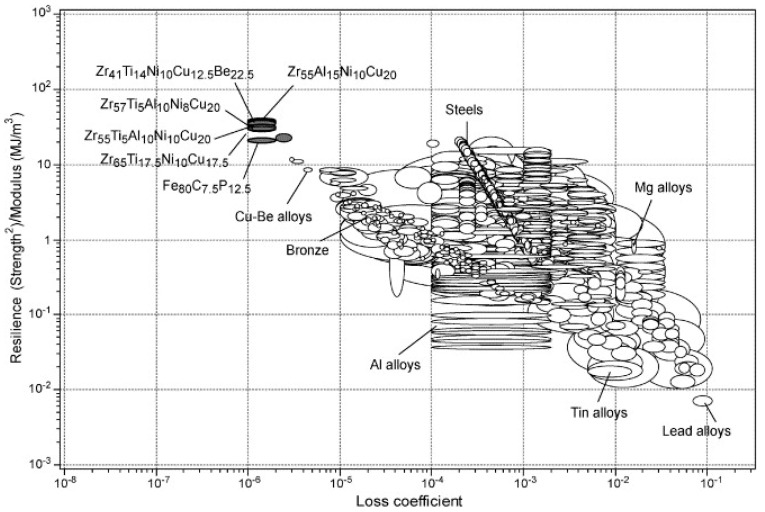
Resilience, *σ_y_*^2^*/E* and loss coefficient for the materials presented in Figure 3 (Reproduced with permission [91]).

According to the discussions in the previous section, there are no crystallographic defects (e.g., dislocation slip systems) to mediate plastic deformation in MGs. Consequently, they exhibit ultrahigh yield stresses closer to their theoretical strength [5]. On the other hand, the elastic moduli of the atomic nearest-neighbour shell in MGs are similar to those in the corresponding metallic crystals [5,89]. However, topological instabilities occur under applied stress due to absence of long-range order, which leads to local anelastic deformations. These anelastic deformations appear macroscopically as lowered stiffness, i.e., a smaller Young’s modulus, *E*, than the corresponding crystalline counterpart [89]. As a result, MGs have an unusual combination of high yield stress, *σ_y_* and low E. In Figure 3, mechanical properties of several representative MGs are compared with more than 1500 engineering alloys and other materials. This figure also presents the contour lines for the elastic strain limit, *σ_y_/E*, which is remarkably high at about 2.67% deformation for a wide range of MGs [90]. Moreover, the resilience, *σ_y_*^2^*/E*, lines are also presented in Figure 3. They are a measure of mechanical damping, i.e., the material’s capacity to store/restore elastic energy per unit volume. For further clarification, the material’s resilience, which is important, for example, in springs, is presented separately in Figure 4. Indeed, higher resilience correlates with a lower loss coefficient in cyclic elastic loading. The loss coefficient reflects the contribution of the local plastic flow in the energy loss [91]. It can be seen that metallic glasses are exceptionally resilient materials.

The high elastic strain and resilience of MGs makes them attractive for various applications, such as elastic pressure gauges, flow-meters, sporting products, and even nanoscale applications [5,92,93,94]. Furthermore, the combination of their superior resilience and low loss coefficient makes them promising for vibrating-reed systems, such as fast-acting springs, gyroscopes, and elastic wave transmission [91]. It has been shown that the elastic strain limit of submicron-sized MG specimens is about twice higher than the already-impressive elastic limit of the bulk samples with the same composition [95]. This is a promising property that can be exploited for MGs in cardiovascular stents having submicron-thick struts. A detailed and comprehensive review on the general elastic properties and corresponding models for a wide range of metallic glasses has been provided by [96].

### 5.3. Plastic Properties: Severe Localized Plasticity

**Figure 5:**
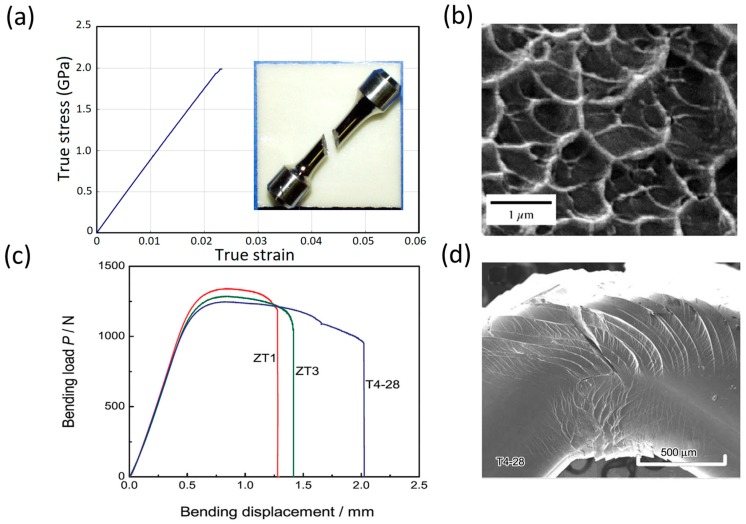
Ductility and failure of MGs under different loading conditions at room temperature (far below *T_g_*). (**a**) A typical tensile fracture in which the plastic deformation is severely localized within a narrow single band and the MG exhibit almost no tensile ductility. Approximately 2% of the elastic elongation is observed. The inset shows the result of catastrophic failure of an MG sample upon tensile loading via the formation of a single shear band aligned at about 45° with respect to the loading direction; (**b**) in contrast with the brittle-like style of stress-strain curve shown in (**a**); the fracture surfaces of MGs display a typical vein morphology typical of “ductile” fractures; (**c**) bending loading test of MG samples, showing extensive bending ductility for different compositions; and (**d**) a prototypical Zr-based BMG sample could exhibit an extensive plastic deformation via multiple shear banding in bending tests. Regardless of overall observed ductility in (**c**); the plastic deformation in this case is still severely inhomogeneous (Adapted with permission [11]).

While the lack of dislocations and slip systems in MGs render them exceptional yield strength and elastic properties, it also results in undesirable effects on their post-yield plastic deformation. At relatively low temperatures respecting their glass transition temperature (*T_g_*), MGs exhibit almost zero tensile ductility and catastrophic failure upon yield, similar to inherently-brittle materials, such as typical silicate glasses [15,16]. However, tensile failure of MGs is accompanied with certain fractographic features different from the silicate glasses as the following [97]:

- The tensile fracture surface of MGs occurs along the plane with maximum resolved shear stress, i.e., at about 45° with the tensile loading axis, in contrast with 90° in typical brittle materials (see Figure 5a).
- The fracture surface of MGs shows ductile features such as “vein patterns” or “river patterns” [2,98,99] (see Figure 5b).

The lack of macroscopic tensile ductility (similar to silicate glass), yet exhibiting ductile fracture surface features (different from silicate glass), in MGs has been attributed to severe localization of plastic deformation in microscopically-narrow bands called shear bands (SBs) [15,16,100,101,102,103,104,105,106]. To describe the origin and mechanism of shear band formation and heterogeneous plasticity in MGs, several theories have been suggested [11,12,13]. These theories are mainly based on two atomistic mechanisms. One is the “free volume mechanism” which proposes deformation-induced dilatation and local softening in the amorphous structure of MGs [107,108]. The other atomistic mechanism is based on local shear transformations and proposes local and cooperative shearing of atomic clusters under applied deformation [109,110]. Once a local region is plastically sheared at a high applied stress, it becomes softer than its un-sheared surrounding regions. Consequently, it becomes more susceptible to subsequent plastic flow. This leads to a spontaneous and auto-catalytic concentration of plastic strains into localized shear-bands [12]. In particular, according to the theory of Argon [109], such shear transformations are the fundamental units of plasticity in MGs. Operation of a shear transformation zone (STZ) to accommodate a certain amount of applied shear strain introduces a localized distortion in the surrounding atoms as well. This distortion in turn, may activate other STZs. Such an autocatalytic process of formation and propagation of shear strain would lead to self-assembly of large planar bands of shear transformation zones, i.e., the shear bands (SBs). Hence, during the tensile test, plastic deformation is severely localized and a single SB readily nucleates. In the absence of any strain hardening mechanism, plastic deformation continues, which may even lead to crack formation within the single SB. As a result, the local shear strain within a single SB is very large (commonly in excess of 100%). However, it is severely inhomogeneous inside a very thin band about 10 to 20 nm thick [111], and the overall macroscopic ductility is negligible.

A lack of tensile ductility is a ubiquitous behaviour in macro-size metallic glasses (either conventional or bulk MGs). In contrast, studies have shown that the behaviour of BMGs in compression and bending tests is more complicated and to some extent, the samples might exhibit macroscopic ductility [112,113,114]. This is mainly due to complex stress state and/or the geometrical confinement of the sample in these experiments. For example, Figure 5c, shows extensive bending ductility for three different BMG samples during a bending test [112]. While these BMGs still do not exhibit any tensile plastic elongation, such bending/compression ductility must be avoided for design purposes. Figure 5d clearly shows that the observed “macroscopic bending ductility” in Figure 5c is a result of extensive formation of multiple shear bands, and still is inhomogeneous/localized [112,113].

Since the focus of this article is on biomedical applications of metallic glasses, we limit our discussions to their mechanical properties at ambient temperatures which is well below their glass transition temperature, *T_g_*. Elevated temperatures, i.e., *T* > 0.6 *T_g_*, impose drastic effects on the mechanical properties of MGs and may lead to extensive homogeneous plastic deformation even in tension [83,115,116]. For a review on the mechanical properties of MGs at a wide range of temperatures, and a deformation mechanism map (as a function of temperature and strain rate), the reader is referred elsewhere [12]. Furthermore, here it is noteworthy that the lack of tensile ductility at ambient temperature is the ubiquitous feature of MG samples at the macro scale, which would be essential for biodevice design and development. At the nanoscale, however, the glassy specimens exhibit high levels of tensile ductility, which is a current subject of extensive research [14,88,117,118]. Besides, in recent years, an innovative and advanced family of MGs composed of nano-sized grains of metallic glasses, i.e., nanoglasses, has emerged exhibiting considerable tensile ductility which is the subject of current theoretical and experimental investigations [119,120,121,122,123,124,125].

### 5.4. Fatigue Properties

As indicated earlier, strength levels in most conventional and bulk metallic glasses approach theoretical levels based on calculations typically used to estimate the theoretical strengths of crystalline metals (on the order of G/10 to G/6). Thus, increases in shear modulus correlate with an increase in strength (see Section 5.1). It is also generally accepted that the high cycle fatigue (HCF) behaviour of crystalline metals typically scales with the strength level [126], although the scaling factor ranges from 0.4–0.5 UTS depending on the crystal structure, i.e., face-centred cubic (FCC), body-centred cubic (BCC), etc., and the important effects of various knock down factors, such as surface roughness, inclusions, porosity, etc. Based on these simple generalizations, it might be expected that BMGs should exhibit excellent HCF behaviour and high fatigue/endurance limits. However, other recent reviews on the stress-controlled fatigue behaviour of BMGs has shown that their fatigue/endurance limits are often much below the generally accepted scaling laws used for crystalline metals and typically fall in the range of only 0.1–0.2 UTS [9,11,12,127,128,129,130]. The source(s) of such poor HCF performance in both drop-cast BMGs [127,128,129,130], as well as melt-spun ribbons [131,132,133], in part relates to process-induced defects (e.g., porosity, isolated crystalline regions), as well as any surface roughness effects in melt-spun ribbons. Such features will severely compromise the HCF behaviour of components intended for use in situations that expose the part to cyclic stresses. Thus, preparation of BMG samples/parts that will minimize such defects will be critical to potentially achieving acceptable performance in any HCF-critical components.

The above review indicates that removal of process-induced defects should improve the HCF performance, and this has been demonstrated [134,135,136]. However, there may be other contributing factors to the generally-low HCF performance, as indicated in recent HCF work by El-Shabasy and Lewandowski [137]. Computational modelling and atomistic simulations have also explained the influence of different parameters on the fatigue behaviour of metallic glasses, such as size effect, etc., [138,139]. Various studies have shown that localized deformation in metallic glasses will occur well below the ‘yield strength’ of such systems in locally ‘soft’ regions of the amorphous material [73,83,109,140,141,142]. These are postulated to act as pre-cursors to eventual macroscopic shear banding and could contribute to the generally-low HCF behaviour. Such localized deformation at stresses well below the UTS will contribute to damage accumulation that will eventually lead to crack initiation and early failure (i.e., at low stresses with respect to the UTS) in metallic glasses [83]. While such localized deformation may contribute to early flow that eventually leads to shear banding/crack initiation, recent work by Schuh et al. [143] has indicated that it might be possible to ‘deactivate’ such sites. Based on this work, via microhardness testing, the microhardness of a BMG sample exhibits a range of values, suggesting that such testing systematically samples locally ‘softer’ and ‘harder’ regions of the BMG. The authors have further showed that repeated indentation of the sample could remove (i.e., deactivate) the ‘softer’ regions and produce a systematic shift in the microhardness to higher values, as well as effectively increase the Weibull modulus. This approach suggests that removal of such ‘softer’ sites could improve the HCF behaviour of BMGs in an analogous manner to fatigue coaxing of crystalline materials [144,145,146,147,148]. Similar effects might be expected by structural (i.e., thermal) relaxation of the BMG, although structural relaxation of BMGs has also been shown to severely compromise the fracture toughness of many BMGs [149].

The work by El-Shabasy and Lewandowski [137] has shown that fatigue cycling of a BMG below the fatigue/endurance limit of a Zr-based BMG tested in 3PB can be used to ‘coax’ the fatigue limit to much higher values. In this regard, the authors have proposed that it might be possible to locally structurally relax the BMG via mechanical means and thereby avoid the negative effects (i.e., reduced toughness) generally obtained in thermally-relaxed BMGs. This work [137] and others [130] has shown that thermal structural relaxation can also improve the HCF behaviour; however, this will occur at the expense of reduced toughness.

While additive manufacturing techniques provide design flexibility, recent reviews [150,151] and works [38,39,152], as well as our summary provided in this section, have shown that process-induced defects (e.g., porosity, LoF, etc.) are often present in as-deposited materials, including BMGs. These defects will need to be removed/eliminated in order to improve the HCF performance. Although applying hot isostatic pressure (HIP) may be possible to close such defects, as shown in many works on crystalline materials [153], the thermal exposures used in HIP may also structurally relax and/or crystallize the BMG and this can severely compromise both the HCF and fracture toughness. Thus, focusing on the production of defect-free as-deposited BMGs/products will be essential, where post-processing may be beneficial to mechanically structurally relax the local regions.

From a cardiovascular stent application standpoint, during the operational lifetime of a typical stent, it is subjected to a continuous cyclic loading condition at a frequency of approximately 72 cycles per minute (normal heartbeat rate) [154]. The US Food and Drug Administration recommends that a cardiovascular stent must be able to withstand at least 400 million cardiac cycles (equivalent of approximately ten years) through HCF resistance, without eliciting fatigue-related failure. Hence, long-term structural integrity of stents, especially their fatigue behaviour, must be one of the major design considerations. In the HCF regime, where typically more than 10^5^ cycles at low stresses are required for failure, the deformation is primarily elastic [155,156]. If the process-induced material defects, e.g., surface roughness, inclusions, porosity, etc., are minimized by optimization of the processing parameters and engaging in repeated elastic deformation of the material sample to remove the ‘softer’ sites in the BMGs, the HCF behaviour of BMG-based stents could be improved. Once such material quality enhancements are carried out, the applied stresses induced by the cyclic physiological loads in the cardiovascular system would not lead to fatigue-induced failure in stents.

### 5.5. Size Effects on Mechanical Properties

As reviewed above, metallic glasses at the bulk scale often exhibit attractive mechanical properties, such as high strength, resilience, hardness, and wear resistance, but typically fail in bulk tension experiments with zero global plasticity. While bulk properties such as strength and plasticity/toughness can be tuned via changes to elastic constants such as the shear modulus (i.e., for strength) [157] and the Poisson ratio (i.e., for plasticity/toughness) [158], recent works have also revealed important effects of sample size and preparation/testing techniques. For example, novel processing techniques [159,160,161,162] have illustrated that MG parts (<1 cm down to the sub-micrometer) may retain the superior properties while improving other behaviours (e.g., plasticity). In contrast to the low/zero tensile plasticity of bulk components [117], thin metallic glass films, pillars, and wires can exhibit outstanding plasticity, depending on processing and sample preparation/testing details [159], together with strengths approaching theoretical values. This aspect is very important for the potential applications of metallic glasses in coatings, wires etc., especially for biological applications. The following provides a short review of size effects on the mechanical properties of metallic glasses. 

#### 5.5.1. Size Effects on Yield Strength and Elastic Strain Limit

As explained in Section 5.1 and Section 5.2, at the bulk scales in most of MGs, the uniaxial yield stress, *σ_y_*, and corresponding yield strain, *ε_y_* (elastic strain limit), are at the level of GPa and ~2%, respectively [95]. Although the *σ_y_* and *ε_y_* of MGs are superior to the conventional crystalline alloys, they are still far from the ideal limit of the materials [91]. As discussed in Section 5.3, the yielding mechanism in MGs at temperatures well below their glass transition temperature, *T_g_*, is due to severe localization of plastic deformation within narrow shear bands [15,101,141]. Shear bands generally nucleate from stress concentrations that are present as casting porosities/flaws, surface cracks, etc., in as-fabricated bulk MGs. In addition, there may be internal “defects” which might readily undergo shear transformations and eventually lead to SB formation [15,101]. Removing such extrinsic structural flaws and local nucleation sites can lead to higher strengths and elastic limits in MGs. Experimental results have been reported that show the benefits of reducing the size of the MG samples to the micron and submicron scales on the enhancement of elastic strain limit and the corresponding stress [95]. The reported elastic strain limit in submicron-sized metallic Cu–Zr MG specimens is about 4.4% and is very close to the predicted theoretical limit of ~4.5% for this material [95]. This suggests that the extrinsic flaws that concentrate stresses in bulk samples have been eliminated in the small-scale sample while also reducing the population of internal structural defects. Consequently, catastrophic shear banding that propagates across a bulk sample as the result of the cooperative activation of microscopic shear events, can be delayed [95]. Furthermore, if the size of such cooperative events approaches the sample dimensions, uniform flow, and extreme ductility, ductile rupture [159] may be promoted, as shown elsewhere and discussed below. 

#### 5.5.2. Size Effects on Ductility and Toughness

While information on elasticity at small length scales directly reflect the inherent structural characteristics of MGs, the size effect on their post-yield plasticity is also important. At the macroscale (i.e., bulk), almost all metallic glasses exhibit zero global plasticity/ductility upon yielding, which is the result of severe localization of plastic deformation in narrow (~10 nm-thick) shear bands [111]. The tensile stresses present within a shear band propagating under tensile loading rapidly leads to catastrophic failure [117]. This may also occur in compression, depending on the sample aspect ratio [158,163]. Hence, MGs exhibit virtually no tensile (or compressive) ductility at the macroscale [84,117]. However, recent observations of some level of post-yield compressive or bending (bulk) plasticity particularly, in alloys with a low ratio of shear modulus to bulk modulus [149], have been attributed to the activation of multiple shear bands [113,142,149,158,164,165], although such deformation is inherently localized.

In contrast, numerous recent studies have demonstrated a brittle-to-ductile transition (which might lead to necking to a point) in the mechanical behaviour of MGs by reducing the sample size down to the nanoscale [88,118,166], although this can depend on both sample preparation and test techniques [159]. In particular, tension or compression tests of nanopillars and thin films [88,95,118,167,168,169], transmission electron microscopy (TEM) tests [166,170,171], and molecular dynamics simulations [84,103,172] have shown that the nano-sized samples undergo homogeneous plastic deformation after yielding. A catastrophic shear-to-ductile rupture transition has been documented [159], while completely different behaviour has been obtained on the identical material after different processing/sample testing techniques. Such nanoscale MGs might exhibit some sort of crack insensitivity [173,174] or sensitivity [82], depending on the chemical composition and the intrinsic ductility of the alloys, as well as the sample preparation/testing details.

The critical thickness/diameter below which the brittle-to-ductile transition (and the consequent necking down to a point) emerges is on the order of 100 nm [88,118]. However, the variations in this critical thickness, and the extent of the plastic extensibility, depend on the intrinsic ductility of the alloy, as well as the extrinsic parameters of such fabrication techniques [73,84,159,167]. For example, while almost no plasticity (<1%) was reported for sub-100 nm moulded Pt_57.5_Cu_14.7_Ni_5.3_P_22.5_ [175,176] and electrodeposited Ni_80_P_20_ [177], there are reports for plastic strains of 23–45% 100 nm copper-moulded Zr_52.5_Cu_17.9_Al_10_Ni_14.6_Ti_5_ [88], 25% true strain for 100 nm-sized Zr_35_Ti_30_Co_6_Be_39_ [118], >150% true strain in sputtered Zr–Ni–Al MG nanopillars with a diameter of ~150 nm [84], and up to 15% true strain for Zr_65_Ni_35_ sputtered thin film with a thickness of 500 nm [169]. While a catastrophic shear-to-ductile rupture transition was obtained in drawn wires, FIB machining of wires to identical dimensions only produced catastrophic shear [159].

Regarding the intrinsic properties of alloys, Lewandowski et al., have found that the intrinsic ductility and toughness of bulk MG alloys can be categorized by the *μ*/*B* ratio, where *μ* is the shear modulus and *B* is the bulk modulus [149]. Alloys with *μ*/*B* < 0.41–0.43 are ductile; otherwise they are brittle. A lower *μ*/*B* ratio (or, equivalently, a higher Poisson ratio) indicates a relative ease of shearing over dilatation, and enhanced the plasticity/toughness [149,178]. Further research at the nanoscale has confirmed that the critical diameter below which the brittle-to-ductile transition occurs is larger for MGs with lower *μ*/*B* ratios (i.e., intrinsically more ductile glasses) [167]. Experiments and atomistic simulations have revealed that higher ductility (i.e., lower *μ*/*B*) in MGs corresponds to a larger plastic process zone size and thicker shear band [179,180]. 

In addition to the intrinsic properties of alloys, the contributions of extrinsic factors to size effects must be also considered. This helps to produce consistent and reproducible data by avoiding artificial/extrinsic effects in experiments using extremely small MG samples. One of the factors introduced by Gao et al. [181] is the shear-band instability index (SBI) in uniaxial compression, which is proposed as proportional to the sample size and inversely related to the machine stiffness. Larger SBI values produce catastrophic failure through the formation of one dominant shear band, while smaller SBI values produce the stable formation of a dense network of shear bands and, hence, a more uniform plastic flow [181]. Other artificial effects may be introduced via the use of focused ion beam (FIB) techniques in the preparation of nanoscale MG specimens [159,182]. Implantation of Ga^+^ ions via FIB machining may produce extra free volume and soften the MG samples [37], while embrittlement via FIB processing/machining has been shown by others [159]. The experiments and molecular dynamics (MD) simulations have also revealed the process/post-process dependence of mechanical properties of MG nanopillars [84,183]. MD simulations have also shown that surface roughness can impart tensile ductility to the nanoscale MGs while increasing the critical thickness of the brittle-to-ductile transition [172]. Further experimental and simulation studies on nanoscale brittle MGs (e.g., FeP and NiP with large *μ*/*B* ratio) have demonstrated that introducing a surface crack to the nanopillars alters the failure mechanism from shear banding to crack propagation [82]. Size effects on fracture toughness are related to the size of the plastic/process zone with respect to the sample thickness and whether plane stress or plane strain conditions prevail [184].

#### 5.5.3. Size Effect on Fatigue Properties

As discussed in Section 5.4, metallic glasses at the bulk size exhibit fatigue/endurance limits often below the generally-accepted scaling laws [9,11,12,127,128,129,130]. The source(s) of such poor high cycle fatigue (HCF) performance is partially related to the process-induced defects (e.g., porosity, isolated crystalline regions) as well as any surface roughness effects. However, MD simulations have indicated that local shear events (i.e., shear transformations) may operate well below the ‘yield’ strength. De-activation of such events via mechanical means [143], via fatigue coaxing experiments [137], or structural relaxation [11,137] may be beneficial to the fatigue performance at the expense of toughness.

On the other hand, the strength, elastic strain limit, ductility, and toughness of MGs may be enhanced by size reduction to the micron- and submicron-scale (see the previous sections under Section 5.5). For example, micropillars with diameters of 3.8 to 0.7 μm exhibited 25–86% higher yield strength than bulk specimens, attributed to a lower defect population in the smaller-sized samples [185]. Thus, reducing the sample size down to the micro- and nano-scale may enhance the HCF properties. Interestingly, recent experiments have revealed that 1.6 μm diameter Zr-based MG samples survived 40 × 106 cycles, and the fatigue limit increased to more than 110% of the bulk yield strength under compression–compression testing, and up to 90% of the bulk yield strength under bending fatigue [186]. Recent large-scale atomistic simulations and finite element simulations of tension-compression fatigue tests in MGs have quantitatively confirmed that small sample sizes should enhance the fatigue life, while large sample sizes should promote cyclic softening and failure [138,139]. These simulations have suggested that shear band thickening is the inherent fatigue mechanism that operates at the nanoscale. The major difference in fatigue mechanisms proposed for MGs tested at the macroscale or nanoscale relates to whether the shear band forms fully or partially through the cross-section of the samples [139]. In spite of these recent works, further investigations are needed to elucidate the fatigue mechanisms of MGs at both the macro- and nano-scale, as well as to determine potential approaches to improve performance based on such an understanding.

#### 5.5.4. Implications of Size Effects on Applications

Advanced technological applications, such as micro- and nano-electro-mechanical systems (MEMS/NEMS), implants and biomedical devices, micro-robotics, etc., have led to increased demands for fabricating materials with desirable properties at miniaturized length scales. However, one major drawback of using conventional crystalline metals in such small-scale applications relates to their anisotropic and/or non-uniform properties when product dimensions approach microstructure length scales. In this regard, a key potential advantage of using monolithic metallic glasses (MGs) in such applications relates to their microscopically-homogeneous behaviour, producing properties that should be isotropic, even at the nanoscale [117]. Thus, it is crucial to understand the mechanical properties of submicron-scale MGs, such as coatings, wires, etc., with potential biological applications. The enhancement of elastic strain limit, yield strength, and fatigue properties is very fascinating from both fundamental and practical reasons and could translate to high (relative) load-bearing applications requiring a high storage of elastic energy, such as in self-expanding cardiovascular stents (see Section 7 and Section 8). 

## 6. Chemical Properties and Biocompatibility

The high corrosion resistance and biocompatibility of MGs can be explained by the absence of structural defects, such as grain boundaries, especially on the surface of metallic glasses, as well as their chemical homogeneity, which act as oxidation sites [6,187]. Such structural and chemical homogeneity allows the formation of a uniform and solid protective layer on the surface of MGs responsible their higher corrosion resistance as compared with their crystalline counterparts [6,187]. The composition of the MGs also has an important role in their corrosion resistance. For example, it is known for a long time that P, Mo, and Cr improve their corrosion resistance [188]. Further studies on the effect of chemical composition and environment have been done by Inoue et al. [33]. MGs containing Nb and Ta alloys exhibit very high corrosion resistance in NaCl and HCl solutions. As an additional element, Nb has the largest effect on the corrosion resistance, followed by Ta, Ti, and Cr [6]. Very recently, magnesium-based BMGs have received significant attention as a new biodegradable material presenting several superior properties compared to their crystalline counterparts [189].

Most of the current understanding of biocompatibility of MGs has been obtained through in vitro and in vivo empirical experiments, and observation of the interactions between the MGs and the host tissues and cells [187]. The response induced by materials to different hosts varies from local and systemic inflammation, toxicity, hypersensitivity, and even tumourogenesis. Such hazardous reactions indicate that before approval and clinical translation of materials preceding comprehensive evidence of their safety is required. Accordingly, much attention has been recently devoted and several comprehensive reviews and research articles have recently appeared in the literature, especially on the biocompatibility of metallic glasses [19,20,187,189,190,191,192,193,194,195,196]. Hence, in this article we do not elaborate on the biocompatibility of MGs and keep our focus on their structural properties as there is a lack of a critical review of the mechanics of MGs, which make them suitable for cardiovascular stents.

## 7. Cardiovascular Stents and Their Required Mechanical Properties

**Figure 6:**
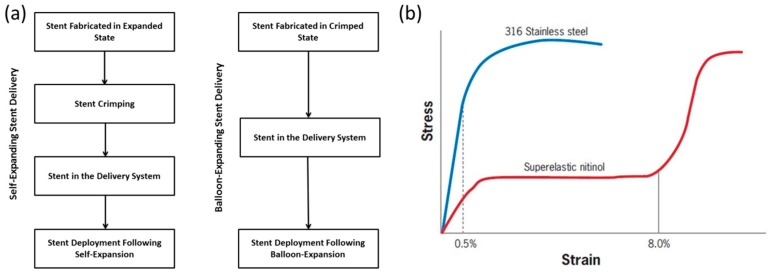
(**a**) Sequence of steps involved in the delivery of self-expanding (SX) and balloon-expanding (BX) stents; and (**b**) a comparison of the stress-strain behaviour of a typical BX stent material (316L stainless steel) and the most popular SX stent material (nitinol).

**Figure 7:**
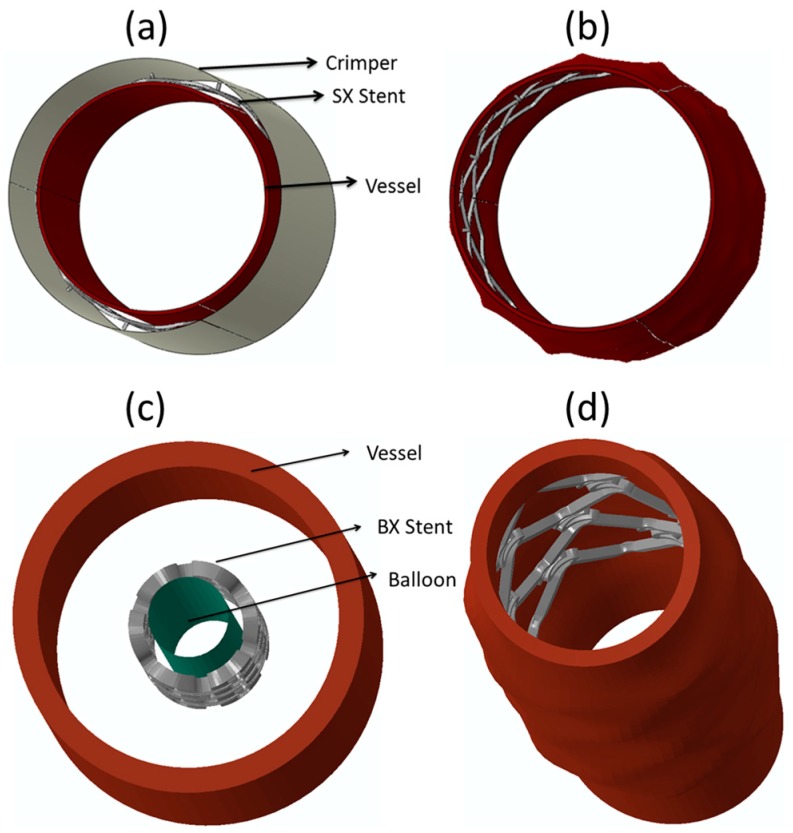
Deployment mechanics of SX and BX stents. (**a**) Arrangement of the crimper, the SX stent, and the blood vessel where the crimper is used to crimp the stent to a much smaller diameter; (**b**) the self-expanded stent in the blood vessel; (**c**) the arrangement of the balloon, the BX stent, and the blood vessel where the balloon is inflated to expand into the blood vessel; and (**d**) the balloon-expanded stent in the blood vessel.

Stents are tube-like structures that are increasingly employed in interventional cardiology revascularization, with proven symptomatic and prognostic effectiveness [197,198,199,200,201,202,203,204,205,206]. They are typically used to treat occlusions, blockages, and aneurysms in endovascular lumen, both in central and peripheral vessels, and also used as bio-prosthetic heart valve frames [154,207,208,209,210,211]. Stents are classified depending on their deployment mechanism either balloon-expanding (BX) or self-expanding (SX) [212]. Table 1 gives the basic differences between BX and SX stents. Figure 6a shows the sequence of steps involved in the deployment of BX and SX stents. SX stents are manufactured and shaped, set at a diameter slightly above the vessel diameter, and are crimped (Figure 7a) and constrained to the smaller diameter until the intended delivery location is reached where, upon removal of the constraint, the stent self-expands (Figure 7b), due to its stored elastic energy. BX stents are either manufactured in a semi-crimped or crimped state [212] and expanded to the vessel diameter by inflating a balloon, wherein it plastically deforms the stent, as shown in Figure 7c,d. 

A BX stent is typically mounted around a balloon, and attached to a catheter which is then maneuvered to the desired site of arterial blockage (typically coronary arteries) and inflated. The balloon inflation process causes expansion of the stent, radially expanding the arterial wall, thus negating the stenosis of the artery [213]. These stents are expected to undergo some degree of permanent plastic deformation in order to secure a good anchorage in the artery [214,215,216]. The plastically-deformed stent is left in place to keep the artery open and to restore blood flow. Stent design and stent material composition plays an important role in the functional efficiency and performance of stents post implantation [217]. 316L stainless steel (Figure 6b) has been used as a balloon-expandable stent material [213,218] owing to its excellent mechanical properties, corrosion resistance, and biocompatibility [219,220,221,222]. Nonetheless, this material possesses some inherent limitations like radiopacity [223], stainless steel allergy [224] in some individuals, and restenosis due to thrombosis [225,226]. It should also be noted that, contrary to what is believed, stainless steel may not be the most inert material [227]. Inflammatory and allergic reactions to metal, particularly due to nickel toxicity, have occurred in patients with orthopaedic, dental, and cardiovascular stainless-steel implants. Research has shown that coronary in-stent restenosis might be triggered by contact allergy to nickel, chromate, or molybdenum ions released from stainless steel stents [227].

SX stents are typically used in clinical conditions where undue radial force that may be exerted due excess balloon expansion is not desirable. After crimping and mounting on a catheter and inserted into the blood vessel, SX stents are released by the delivery system to self-expand and exert a radial force on the blood vessels to keep them open [200]. The stents self-expand into the vessel until stress equilibrium is reached between the vessel and the stent, where the stent is in compression and the vessel in tension [212,228]. The extent of expansion and magnitude of the outward force exerted by the stent on the vessel can be controlled through the stent design and choice of stent material. Efficient deployment of an SX stent will depend on whether the stent undergoes static failure during crimping, where a relatively larger stent is compressed and mounted on the smaller catheter [229]. A failure during crimping will hamper the deployment process. Thus, crimping and deployment are equally important to determine the final outcome of the actual surgical procedure.

Currently, nitinol (Figure 6b) is the most popular choice as the material for SX stents [230,231,232,233,234]. Contrary to other conventional engineering materials, fracture in a nitinol based SX stent is not stress-based but strain-based [235]. In general, one of the most important mechanical properties required of an SX stent material would be the high recoverable strain limit during stent crimping (often referred to as crimping strain) [207,229]. Nitinol offers a high recoverable strain (Figure 6b) of about 8–10% [236,237,238] due to austenite-martensite-austenite phase transformation. This transformation induced the high recoverable strain of nitinol, reducing the risk of failure of the stent, especially during crimping where the stent is bound to undergo large deformations. Clinicians have often capitalized on this important property to crimp stents to smaller profiles during percutaneous procedures. However, nitinol has some limitations that include the high material processing cost [239], the complexity to machine nitinol-based stents [240,241,242], and phase transformation-induced complications. Nitinol is quite an expensive material [241] owing to extensive processing costs and a very limited number of suppliers. Cost has often impeded the utility of nitinol in highly-competitive and/or cost-driven applications. The rise of health care service expenditure, including stringent reimbursement policies by insurance companies in a large number of advanced countries may discourage the use of nitinol unless the nitinol-based device is a critical requirement for the therapy. Nitinol’s non-linear and complex behaviour with thermal dependency [241] are presenting a variety of design challenges add to the material processing complexity [243]. The tooling can be complicated and critical heat treatment parameters can drastically alter the mechanical properties of the material like transformation temperatures, residual stresses, etc. Nitinol undergoes phase transformation in its crystal structure [244] when cooled from the austenite to martensite phase [245]. This inherent phase transformation is the basis for the unique and extremely useful properties of these alloys; which are shape memory and superelasticity. When a shape memory alloy is in its martensitic form, it can be easily deformed into any shape. However, when the alloy is heated through its transformation temperatures, it reverts to austenite and recovers its previous shape due to the high transformation strain. This process is known as shape memory. Nitinol also exhibits superelastic behaviour when deformed at a temperature slightly above its transformation temperatures. This effect is due to the stress-induced formation of martensite above its normal temperature. As it has been formed above its normal temperature, the martensite reverts immediately to the undeformed austenite as soon as the load is removed [238,240,241,244,245]. Though shape memory and superelastic properties have been explored by clinicians and biomedical scientists to design nitinol-based stents, the above-mentioned phase transformations pose some complications. For example, a nitinol-based stent is crimped at a low temperature where it is martensitic, and mounted onto a catheter. However, it is then stored at room temperature where there is a temperature-induced transformation from martensite to austenite, and yet the stent is not allowed to recoil as it is sheathed. This may lead to additional residual stresses in the stent, which may affect its deployment once inserted into the body, and may also affect its fatigue endurance when subjected to pulsatile physiological loads. A recent review has also shown significant scatter in the fatigue performance of nitinol wires [246].

Hence, due to the inherent limitations of stainless steel and nitinol, there is a quest for new materials with equally good mechanical properties which could eventually be an alternative for these existing materials. BMGs are being evaluated as potential cardiovascular stent materials, and finite element method (FEM) is being used to model BMG-based stents [20,21]. A recent work reported some interesting FEM based results comparing BMG and 316L stainless steel as stent materials [21]. However, as described in the previous sections of this review, BMGs are brittle and undergo catastrophic failure through shear banding beyond their yield point. Hence, BMGs cannot replace stainless steel in balloon-expanding stent applications as the main requirement of such stents is the homogeneous and permanent plastic deformation beyond the yield point. In contrast, and interestingly, for self-expanding stent applications, BMGs could be tapped successfully as they offer superior mechanical properties, such as large elastic strain limit, high strength, and resilience (i.e., high elastic energy storage), improved wear resistance, fatigue endurance, and excellent high temperature formability, in comparison to crystalline metals. Furthermore, using BMGs there is no requirement for temperature induced phase transformation, and the stent expansion is simple elastic recoil of the crimped stent when the load is removed. Table 2 gives the comparison between nitinol and BMG as a self-expanding stent material.

## 8. Application of Metallic Glasses in Self-Expandable Stents

According to our discussion so far, the superior resilience of MGs makes them very promising for use in SX stent applications. Particularly, the elastic spring-like restoring of the MG-based SX stents is arguably a significant improvement on nitinol-based devices in this category of stents. However, the major weakness of MGs is their near zero tensile ductility which makes them susceptible to catastrophic failure beyond their elastic limit. In relation to this, we have recently demonstrated that, by taking this major caveat into account, it would be possible to exploit the high resilience of BMGs in SX stent applications. In particular, we investigate the performance of such stents during the crimping and deployment processes as reported in the literature.

In our recent work [247], we aimed to study the mechanical behaviour of a prototypical Zr-based BMG during crimping of four SX stents designed for percutaneous applications, including percutaneous carotid artery stenting (CAS), transcatheter aortic valve implantation (TAVI), percutaneous cava valve implantation, and transcatheter mitral valve replacement (TMR). The Zr-based BMG material model was a linear elastic material with a Young’s modulus of 80 GPa and a Poissons’s ratio of 0.38 [247]. 

**Figure 8:**
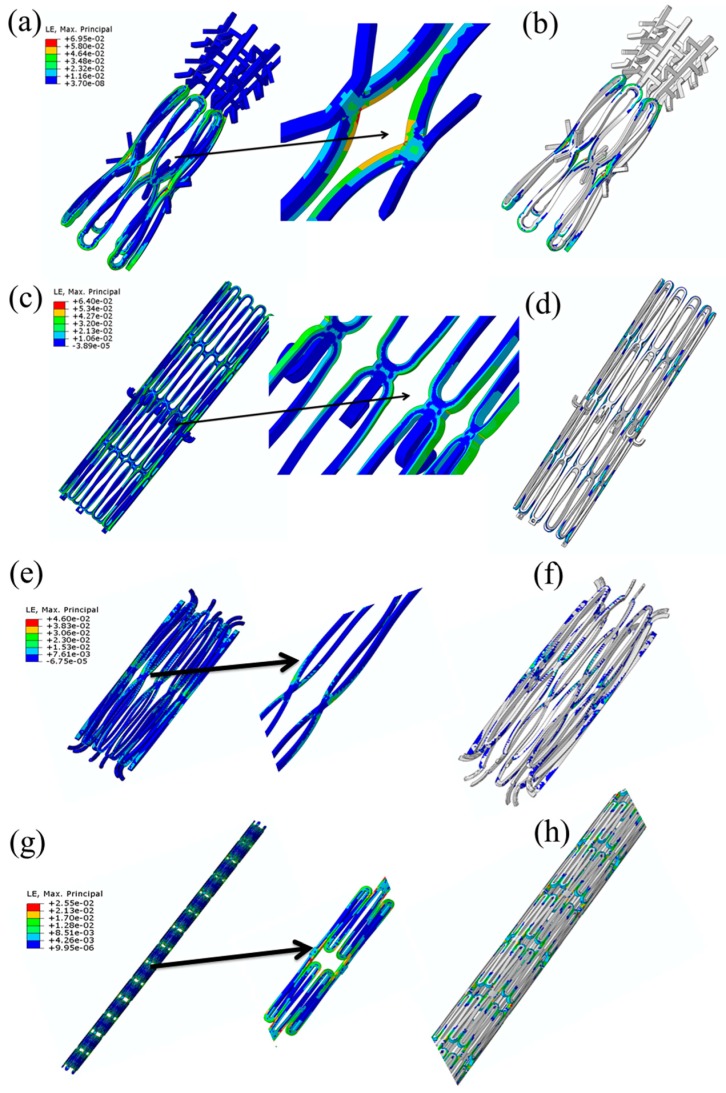
Finite element simulation of diamond-shaped stent crimping. (**a**) Maximum crimping strain in the mitral stent after crimping to 18F; (**b**) the critical locations in the mitral stent that would fail upon loading beyond 2% elastic elongation (colorful regions); (**c**) the mMaximum crimping strain in the caval stent after crimping to 24F; (**d**) the critical locations in the caval stent that would fail upon loading beyond 2% elastic elongation; (**e**) the maximum crimping strain in the aortic stent after crimping to 18F; (**f**) the critical locations in the aortic stent that would fail upon loading beyond 2% elastic elongation; (**g**) the maximum crimping strain in the Protégé stent after crimping to 6F; and (**h**) the critical locations in the Protégé stent that would fail upon loading beyond 2% elastic elongation (reproduced with permission [247]). The French scale or French gauge system is commonly used to measure the size of a catheter [249,250]. It is usually abbreviated as Fr or FR, or simply F. The French size is three times the diameter in millimetres [251]. For example, if the French size is 18, the diameter is 18/3 = 6 mm.

**Figure 9:**
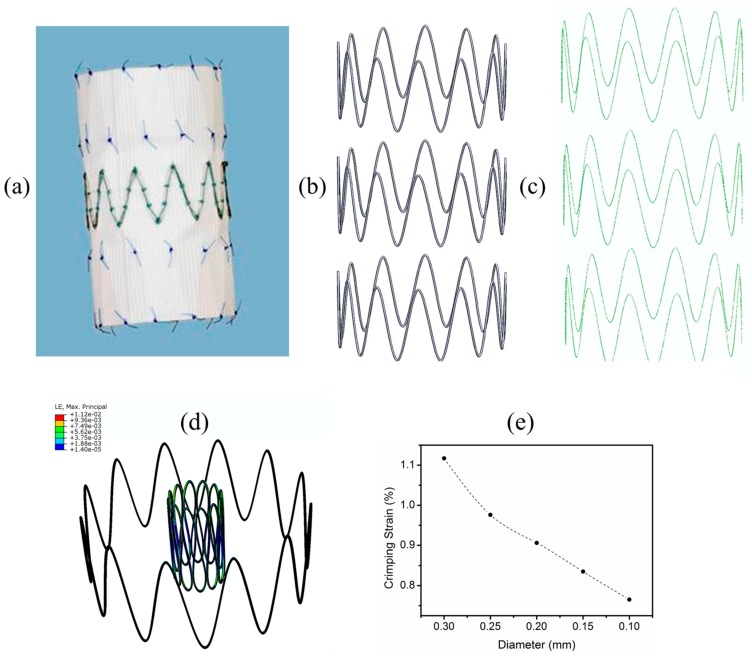
3D CAD Models and finite element simulation of Zenith stent crimping. (**a**) Zenith TX2 TAA Endovascular Graft [248]; (**b**) an isometric view of the CAD model of the bare metal wire based stent; (**c**) the stent meshed with beam elements; (**d**) maximum crimping strain in The Zenith stent after crimping to 22F; and (**e**) a crimping study showing the impact of stent wire diameter on the crimping strain. (Reproduced with permission [247,252]).

Figure 8a,c,e,g show the maximum crimping strains of 6.95%, 6.06%, 5.17%, and 2.55% in the mitral, caval, aortic, and the Protégé stent, respectively. It was found that the locations where the strain values exceeded 2% (nominal elastic strain limit of MGs) were at regions that typically undergo large deformations at the inter-diamond-shaped crown junctions, which is a typical trend observed in stents designed with diamond-shaped crowns [228,236,238]. Figure 8b,d,f,h show the critical locations in the stents where the strains exceed 2% while crimping. In other words, BMGs may not be beneficial as structural materials for the development of stents with diamond-shaped crowns since, during crimping, shear banding and catastrophic failure occur at the external and internal sides of their diamond-shaped crowns. Hence, our findings imply that, regardless of the superior mechanical and functional properties of BMGs, in order to use them as stent materials, it is important to choose applications where the SX stents do not consist of diamond-shaped crowns. One such application is endovascular repair for aortic aneurysm, where a non-diamond-shaped stent is used to develop the Zenith TX2 TAA endovascular graft used to treat aortic aneurysms [248]. The Zenith stent used in our study is shown in Figure 9a–c. We conducted crimping of the Zenith stent to 22F and Figure 9d illustrates that the maximum crimping strain in the BMG-based Zenith bare metal stent is about 1.1% when the stent was crimped appropriately. This crimping strain is below the critical strain limit of BMGs and it could be concluded that shear banding and catastrophic failure was avoided. From Figure 9e it can also be inferred that reduction of the wire diameter from 300 μm to 100 μm results in further reduction of the crimping strain in this stent, which renders the device even safer where static failure during crimping could be avoided further. 

**Figure 10:**
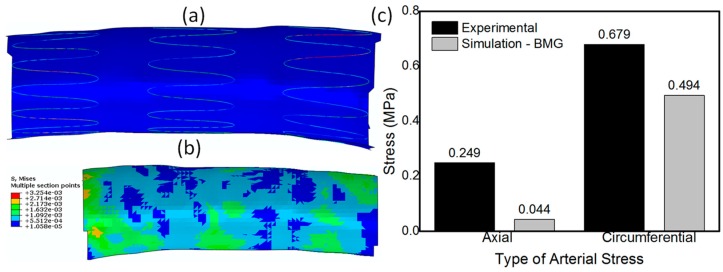
(**a**) During continuum modeling, stents are allowed to expand into the vessel until stress equilibrium is achieved between the stent and vessel; (**b**) a contour plot of von Mises stress distribution (*S*, von Mises) in the post deployment descending aorta when the stent material is BMG; and (**c**) a histogram comparing the axial and circumferential stresses in the artery after stent deployment with BMG as the stent material against experimental test results on the failure strength on human arteries (reproduced with permission [252]).

After we evaluated the feasibility of using BMGs as a stent material for SX stents with non-diamond crowns and ascertaining that the stent would not fail during crimping, we evaluated the safety of its deployment in a patient specific artery. It is to be noted that though stent crimpability is very important, its real-time deployment in a patient specific blood vessel is equally important and is highly impactful on the final outcome of the surgical procedure. Very high arterial stresses and vessel stretching, post-stent deployment could result in profound long-term histological response including exuberant neointimal proliferation and luminal stenosis [253]. For this exercise, we simulated the deployment of a BMG-based Zenith TX2 TAA stent by letting it self-expand in a patient specific descending aorta to quantitatively evaluate the arterial stresses and the vessel deformation due to stent deployment. The boundary and loading conditions used for the deployment simulation has been described in detail in our recent work [252]. Figure 10a shows the SX BMG based Zenith stent deployed in the aorta and Figure 10b shows the contour plot for the distribution of stresses generated in the arterial walls of descending aorta upon stent deployment. The histogram depicted in Figure 10c compares the axial and circumferential stresses in the aorta due to the deployment of the BMG-based Zenith stent with the ultimate failure strength of the human artery. This figure clearly demonstrates that both the axial and circumferential stresses due to the BMG stent deployment are much lower than the failure limit of human arteries. Hence, it can be concluded that, upon careful design, BMG-based stents can be used safely during the crimping and deployment procedures, suggesting that BMGs are promising candidates for future stent applications [21,247,252].

## 9. Summary

To clarify the critical design and performance issues regarding to the application of bulk metallic glasses (BMGs) as structural materials in cardiovascular stents, in this review we provided a brief, yet comprehensive, discussion on the fundamentals of BMGs from a materials science point of view, including the formation, processing, structural features, and the relation between their structure and mechanical/chemical properties. We reviewed the possibility of using additive manufacturing (AM) techniques to fabricate patient-specific cardiovascular stents using BMGs. We further elaborated on the fatigue behaviour of BMGs and the caveats which must be considered in adjusting the AM parameters to avoid undesirable structural defects having destructive effects on the fatigue resistance of the resultant stent products. 

We further discussed different categories of cardiovascular stents, their required mechanical properties, and the metallic alloys currently in use. We explained how the outstanding mechanical properties of BMGs could be promising for potential stent applications. Particularly, the exceptional resilience of BMGs allows them to substantially store higher amounts of elastic energy per unit volume than their conventional crystalline counterparts. Such a property makes them promising to be used as self-expandable stent materials. However, the Achilles heel of BMGs is their near-zero tensile ductility, which makes them susceptible to catastrophic failure beyond their elastic limit which must be considered as a design caveat. We explained that, by taking this major caveat into account, it would be possible to exploit the high resilience of BMGs in self-expandable stent applications. In this regard, we explained our recent findings on the mechanical performance of BMG-based self-expandable stents during the deployment process as compared with the conventional nitinol-based stents. 

We intend to inspire the fraternity of biomechanists, biomedical engineers, clinicians, and medical device manufacturers towards the realization of BMG-based stent applications. 

## Figures and Tables

**Table 1:** Difference between balloon-expanded (BX) and self-expanding stents (SX).

Self-Expanding Stent	Balloon-Expanded Stent
Manufactured in expanded state	Manufactured in crimped state
Self-expansion due to stored elastic energy	Expansion using balloon inflation pressure
Expansion cannot be manually controlled	Expansion is a controlled process
No plastic deformation	Permanent plastic deformation
Used in bigger arteries and as valve replacement devices	Typically used smaller vessels like coronary arteries
Nitinol, shape-memory polymers, etc.	Stainless steel, Co-Cr, Platinum alloys, etc.

**Table 2:** Nitinol vs. BMG as a self-expandable stent material.

Nitinol	BMG
Temperature induced phase transformation	No phase transformation
Super elastic	Purely linear elastic
Crimping should be done only at a low temperature	Crimping can be done at any temperature
Temperature controlled recoil	Spring back-like recoil
Strut thickness based on application	Thinner struts giving superior strength
Difficulty in crimping with thicker struts	Thinner struts aid in better crimpability
Thicker struts may lead to restenosis	Thinner struts reduce rate of restenosis
Associated problems with phase transformation	No phase transformation related problems
